# Evaluation of Different Single-Walled Carbon Nanotube Surface Coatings for Single-Particle Tracking Applications in Biological Environments

**DOI:** 10.3390/nano7110393

**Published:** 2017-11-16

**Authors:** Zhenghong Gao, Noémie Danné, Antoine Guillaume Godin, Brahim Lounis, Laurent Cognet

**Affiliations:** 1 Laboratoire Photonique Numérique et Nanosciences, University of Bordeaux, UMR 5298, F-33400 Talence, France; zhenghong.gao@gmail.com (Z.G.); noemie.danne@institutoptique.fr (N.D.); antoine.godin@mail.mcgill.ca (A.G.G.); brahim.lounis@u-bordeaux.fr (B.L.); 2 Institut d’Optique & CNRS, LP2N UMR 5298, F-33400 Talence, France

**Keywords:** single-walled carbon nanotube, encapsulation, single particle tracking, photoluminescence, bio-imaging

## Abstract

Fluorescence imaging of biological systems down to the single-molecule level has generated many advances in cellular biology. For applications within intact tissue, single-walled carbon nanotubes (SWCNTs) are emerging as distinctive single-molecule nanoprobes, due to their near-infrared photoluminescence properties. For this, SWCNT surfaces must be coated using adequate molecular moieties. Yet, the choice of the suspension agent is critical since it influences both the chemical and emission properties of the SWCNTs within their environment. Here, we compare the most commonly used surface coatings for encapsulating photoluminescent SWCNTs in the context of bio-imaging applications. To be applied as single-molecule nanoprobes, encapsulated nanotubes should display low cytotoxicity, and minimal unspecific interactions with cells while still being highly luminescent so as to be imaged and tracked down to the single nanotube level for long periods of time. We tested the cell proliferation and cellular viability of each surface coating and evaluated the impact of the biocompatible surface coatings on nanotube photoluminescence brightness. Our study establishes that phospholipid-polyethylene glycol-coated carbon nanotube is the best current choice for single nanotube tracking experiments in live biological samples.

## 1. Introduction

Over recent years, the numerous improvements in optical microscopy that allowed robust single-molecule detection has generated novel knowledge of various biological paradigms. In living samples, single-molecule/particle tracking (SPT) gives access to complex dynamic organizations down to the molecular scale [1]. Fluorescence microscopy has been the ubiquitous approach for performing single-molecule detection and SPT experiments because of its high sensitivity, specificity, and spatiotemporal resolution [2]. Most current single-molecule studies are limited to cultured cells [3] or thin tissue preparations [4,5], therefore lacking many important aspects to allow the study of real tissue morphologies and activities. Since biological samples strongly scatter light [6] and display substantial auto-fluorescence at visible wavelengths [7], SPT studies in intact tissues are challenging with most common visible single-molecule probes. Because the biological transparency window lies in the near-infrared (NIR) range where absorption, scattering, and auto-fluorescence are minimized [8], the identification of stable luminescent nanoscale emitters in the NIR is the preferred route toward deep tissue investigations at the single-molecule level.

In this context, single-walled carbon nanotubes (SWCNTs) are unique luminescent probes due to their brightness, photostability, and NIR spectral imaging range [9,10]. In particular, high signal-to-noise ratio imaging of SWCNT ensembles was previously reported in whole animals [11,12]. At the single-molecule level, functionalized SWCNTs have been tracked at the membrane or in intracellular regions of live cells [13,14] while non-functionalized SWCNTs were imaged intracellularly in cultured cells [15], but also in the extracellular space of multicellular tumor spheroids [16] and more recently in acute brain slices [17]. Importantly, most of these studies rely on the encapsulation of SWCNTs by polymers, but the impact of the SWCNT wrapping moieties on eventual non-specific interactions between SWCNTs and living cells has usually been overlooked (see Table 1). Here, non-specific interactions denote, e.g., electrostatic, Coulomb, or van der Waals interactions with cells as opposed to specific and controlled SWCNT-biomolecular interactions. This knowledge is crucial, however, since non-specific interactions between SWCNTs and cells might be at the origin of cellular toxicity. In addition, for many applications, such as live tissue imaging, non-specific interactions between SWCNTs and cells must be minimal in order to allow SWCNT-nanoprobes to access (or circulate) in the complex structures of the probed tissues. Finally, in the context of single-molecule imaging, the choice of the polymeric wrapping agent must also ensure bright and stable SWCNT photoluminescence to allow easy nanotube detection and long-term single-molecule imaging. Indeed, SWCNT photoluminescence is highly sensitive to local environments and the choice of SWCNT encapsulation agents is critical to allow efficient SWCNT photoluminescence detection at the single-molecule level.

Herein, we screened several common coatings for encapsulating carbon nanotubes that have the potential of offering luminescent SWCNTs that display low cellular toxicity and minimal non-specific interactions with cell membranes. We identified the best surface coatings to allow high signal-to-noise ratio detection of single SWCNTs for SPT applications in biological environments.

## 2. Results and Discussion

### 2.1. Cytotoxicity Experiments

We first evaluated the cytotoxicity of SWCNTs encapsulated with different moieties. Although surfactants like sodium dodecylbenzene sulfonate or bile salts are known to provide the best luminescing SWCNTs in aqueous environments [24,25,26], their use for cellular applications should be avoided because these surfactants inherently alter the integrity of cellular membranes. Here we considered phospholipid-polyethylene glycol (PLPEG) [11,27], pluoronic (F108) [15], Tween20 [21,28], Brij35 [29], and in situ polymerized (poly)vinyl pyrrolidone (ISPVP) [23] -coated nanotubes, as they are potential biocompatible surface coatings that can be used to solubilize photoluminescent SWCNTs (see Table 1).

In brief, HiPco synthesized nanotubes were suspended by PLPEG, F108, Tween20, and Brij35 with the following protocols (see Materials and Methods section for further details): for PLPEG preparation, 1 mg of raw SWCNTs and 5 mg of coating molecules were added in 5 mL D_2_O water and dispersed by tip sonication; for F108, Tween20, and Brij35 preparations, 2 mg of raw SWCNTs and 1 wt % coating molecules were mixed in 2 mL Milli-Q water and also dispersed by tip sonication (20 W output for 8 min in an ice bath). Nanotubes bundles and impurities were precipitated by centrifuging the dispersion and the supernatant was collected and stored. For ISPVP preparation, we followed the protocol described in Reference [23]. Before cell incubation, excess coating material was removed by filtering the dispersions through a 100 kDa MWCO (Molecular Weight Cut Off) filter under centrifugation. Purified nanotubes were re-dispersed in Milli-Q water and the final nanotube concentration was adjusted to be 1 μg/mL in the cell culture media, as required for single nanotube detection (i.e., ~10 SWCNTs having typical lengths of 500 nm in 10 × 10 × 10 µm^3^).

For cytotoxicity experiments, we chose a standard cell line in biological laboratories (COS-7 cells, derived from CV-1, a simian cell line Cercopithecus aethiops). In all experiments, the quantity of COS-7 cells was adjusted to 1 × 10^5^ cells/mL for initial incubation with SWCNTs. The final cell number was counted using a white light microscope and dead cells were identified using a trypan blue staining. Cell proliferation and viability were then calculated (see Materials and Methods section for calculation).

**Figure 1:**
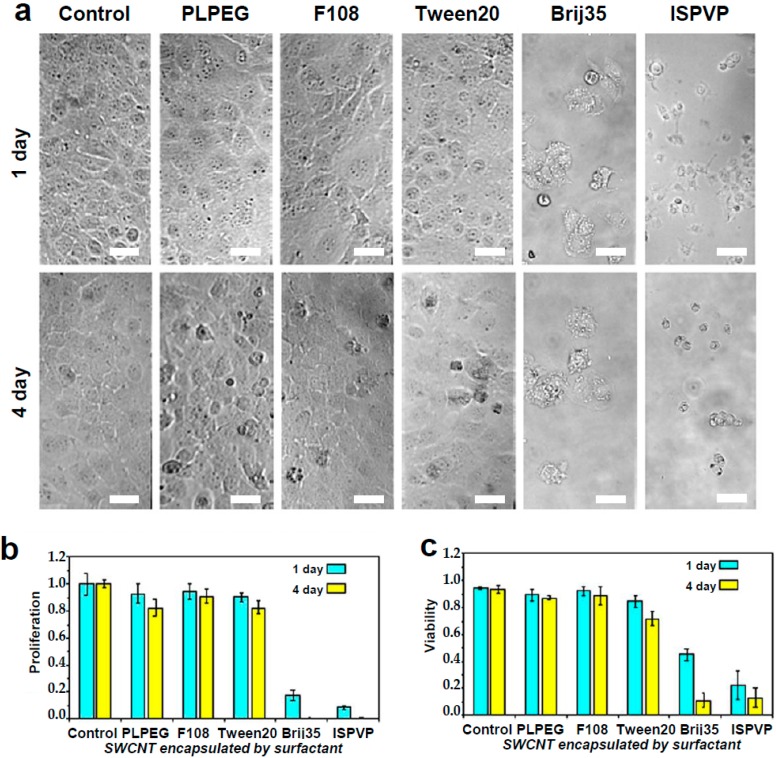
Live cell biocompatibility of SWCNTs encapsulated with different coatings. Top (**a**): Bright-field images of COS-7 incubated with PLPEG-, F108-, Tween20-, Brij35-, and ISPVP-coated SWCNTs for one day and four days. Scale bar: 30 µm. Bottom: Corresponding comparisons of cellular (**b**) proliferation and (**c**) viability. Starting concentration of COS-7 cells: 1 × 10^5^ cells/mL, SWCNT: 1 μg/mL, cell cultured at 37 °C with 5% CO_2_; Three independent experiments were performed to obtain standard variations. Cell viability was evaluated using trypan blue dye staining.

After one day of incubation, the viability and morphology of cells exposed to PLPEG-, F108-, and Tween20-coated nanotubes were very similar to those of control cells (e.g., without nanotube administration) (Figure 1), while after four days of incubation only a small number of dead cells could be observed. For cells incubated with Brij35- and ISPVP-coated nanotubes, the situation was very different since the vast majority of the cells were either detached from the culture plate surface or dead at both one day and four days of incubation (Figure 1). In terms of cell proliferation, PLPEG-, F108-, Tween20-coated nanotubes did not interfere significantly with the cells as compared with control cells, while a dramatic proliferation inhibition resulted from the incubation with Brij35- and ISPVP-coated nanotubes for both one day and four days. These results suggested that in terms of acute cellular toxicity, PLPEG-, F108-, or Tween20-coated nanotubes administrated at up to 1 μg/mL would be preferred for biological applications, while Brij35 and ISPVP coatings must be avoided due to their strong effects on cells.

**Figure 2:**
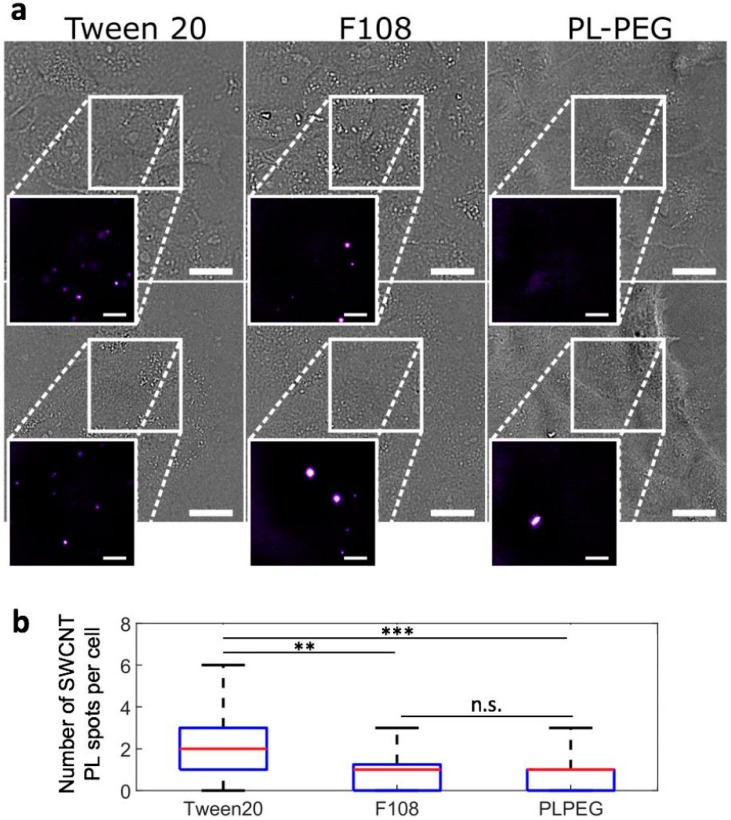
SWCNT interactions with live cells probed by NIR photoluminescence. (**a**) Bright-field and NIR photoluminescence imaging (inserts) of live cells incubated for 24 h with Tween20-, F108-, or PLPEG-coated SWCNTs and further rinsed before imaging. PLPEG- and F108-coated SWCNTs displayed lower non-specific interactions with live cells compared to Tween20-coated SWCNTs. Scale bars: 25 µm for the bright field images and 10 µm for the magnified NIR photoluminescence images of SWCNTs; (**b**) Corresponding median (red), 25–75th percentile (blue), and 0–100th percentile (black) of the number of SWCNT PL spots observed on live cells for Tween20-, F108- or PLPEG-coated SWCNTs (*N* = 70, 53, and 86 cells respectively, n.s.: not significant, ** *p* < 0.01, *** *p* < 0.001, Kolmogorov-Smirnov test).

We then compared by photoluminescence imaging the degree of non-specific interaction of PLPEG-, F108-, and Tween20-coated SWCNTs with live cell surfaces following 24 h of incubation at 37 °C with 5% CO_2_. Before imaging, cells were rinsed one time to remove nanotubes in suspension. Experimentally, non-specific interactions are assessed by the immobilization of SWCNTs on living cells after rinsing, which prevents the nanotubes from freely diffusing in the cell culture medium. A single SWCNT photoluminescence imaging setup was used based on an upright microscope equipped with an Electron-Multiplying Charged Couple Device (EMCCD) camera and a 1.0 NA 60× objective in a wide-field configuration (Materials and Methods). We focused here on (6,5) SWCNTs which emit at ~986 nm, using excitation at 845 nm to efficiently excite (6,5) SWCNTs at a phonon sideband [30], with a tunable Ti:Sa laser with circularly polarized light. Both excitation and emission wavelengths were then in the biological transparency window, which minimizes potential biological tissue phototoxicity from laser or photoluminescence light. We found that after 24 h of incubation followed by medium rinsing, Tween20-coated SWCNTs displayed a significant amount of interactions with the live cells as opposed to F108- and PLPEG-coated SWCNTs (Figure 2). This interaction can clearly be observed here in the Tween20-coated SWCNT cell samples by the presence of several individualized photoluminescent spots, which may also represent some small nanotube bundles. Although it was not observed during the course of our experiments, we cannot exclude that Tween20-coated SWCNTs might eventually be released from the cells to the cell medium after a certain time. Stable interactions might also lead to internalization, as observed with other nanotube encapsulations designed to strongly interact with cells [31]. The results showing that PLPEG-coated SWCNTs do not significantly interact with cells confirmed previous observations [17].

### 2.2. Photoluminescence Imaging of Biocompatible Nanotubes

#### 2.2.1. Photoluminescence Imaging of Biocompatible Nanotubes in Biological Medium

**Figure 3:**
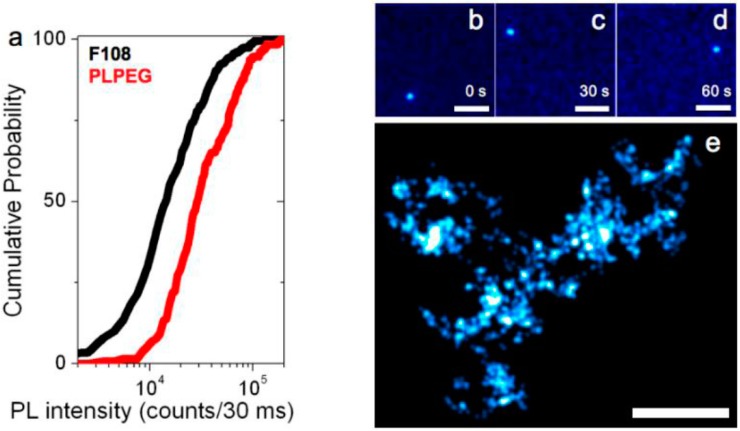
Photoluminescence imaging of individual SWCNTs. (**a**) Cumulative distribution of the photoluminescence intensities from 162 (resp. 256) individual PLPEG- (resp. F108-) coated SWCNTs in biological media (DMEM); (**b**–**d**) Single PLPEG-SWCNT tracking in 1.5% agarose gels: three frames, separated by 30 s, of a ~1 min movie acquired at 33 Hz are displayed revealing the SWCNT trajectory within the gel; (**e**) Super-resolved map of the gel structure reconstructed from the collection of 2096 super-localized nanotube positions while the nanotube was diffusing. Scale bar: 5 µm.

We also quantitatively compared the photoluminescence of PLPEG-, F108-, and Tween20-coated carbon nanotubes at the single nanotube level when immersed in cell culture medium. In standard serum-rich biological medium (Dulbecco’s Modified Eagle Medium (DMEM)), it is found that PLPEG-coated nanotubes are significantly brighter than F108-coated nanotubes at identical imaging conditions (Figure 3). The median brightness of individual PLPEG-coated SWCNTs was indeed 2.1 larger than that of F108-coated SWCNTs (Figure 3a). Tween20-coated nanotubes could not be compared at the single tube level since in the biological medium, nanotube aggregation was frequently observed (not shown). This observation might be due to surface coating instabilities, which might also account for the observed non-specific interactions between SWCNTs and cellular membranes. We also noticed some degree of non-specific interaction of F108-coated SWCNTs at the surface of the microscope glass-slides, unlike the results for PLPEG-coated nanotubes. In fact, although pluronic-coated nanotubes were previously reported to be useful for imaging in cells [19,32], earlier investigations have indicated that F108 molecules may detach from nanotube surface and quickly be replaced by biological serum in the physiological environment [15], and then impact non-specific interaction properties of the corona complex with its surroundings [33]. Such replacements were not reported for PLPEG-coated nanotubes, which could be imaged at the ensemble level in animals [11] and at the single nanotube level in live tissue [17].

In the aqueous phase suspension of PLPEG-coated SWCNTs, the hydrophobic lipid head of PLPEG directly interacts with the carbon nanotube surface via hydrophobic interaction while the hydrophilic polyethylene glycol (PEG) chain is extended in water and stabilize nanotubes. PLPEG encapsulation helps in preserving carbon nanotubes’ pi-conjugation backbone, which is essential for sustaining the nanotubes’ photoluminescence brightness. In biological applications, the soft PEG scaffold layer minimizes non-specific adsorption of various biomolecules [11,34]. In principle, the PEG length and density can be adjusted to further minimize non-specific interaction with cells. In addition, terminal groups can be incorporated in the PEG chain (e.g., –NH_2_ or –COOH) for allowing precise functionalization and offer specific molecular recognition with low non-specific interactions (e.g., by grafting antibodies to the PLPEG-coated nanotubes).

#### 2.2.2. Photoluminescence Imaging of Biocompatible PLPEG- or F108-Coated SWCNT in Thick Biocompatible Aqueous Gels

We finally compared the diffusive behavior of PLPEG- or F108-coated SWCNTs that were found 50- to 100-µm deep within 1.5% aqueous agarose gels by tracking their movements at the single nanotube level at video rate (30 ms integration time per frame). Figure 3b–d shows the photoluminescence images of a (6,5) nanotube coated with PLPEG recorded at three arbitrarily chosen time points separated by 60 s during its free diffusion in the agarose gel. PLPEG-SWCNT luminescence intensity and photostability are found to be excellent within their biocompatible coating allowing recordings over several minutes, similar to the bright bile salts suspended SWCNTs [35]. We note that F108-coated SWCNTs were not diffusing in these gels and individual nanotubes were found immobilized, possibly because of non-specific interactions or steric hindrance due to corona effects [33] following F108 replacement [15]. On the contrary, PLPEG-SWCNT diffusion matches that of SWCNTs coated with bile salts previously studied in similar gels [35]. Finally, owing to the high signal-to-noise ratio at which individual PLPEG-coated SWCNTs can be detected, one can super-localize the nanotube center-of-mass (i.e., determine its position) with precisions of ~50 nm, well below the diffraction limit (equal to ~600 nm for a 986-nm emitting SWCNT detected with a 1.0 Numerical Aperture (NA) objective used here). The collection of nanotube super-localizations during its exploration of the gel structure further provides a map of the gels diffusive environment with 50 nm resolution. In Figure 3e, each localization is displayed as a two-dimensional Gaussian of 50 nm width and unit amplitude as commonly used in localization microscopy [2].

## 3. Materials and Methods

### 3.1. Preparation of SWCNT Dispersions

Pluoronic (F108-), Tween20-, and Brij35-coated SWCNTs: All chemicals were purchased from Sigma-Aldrich (St. Louis, MO, USA) if not stated otherwise. In a typical preparation, 2 mg of HiPco-synthesized SWNTs (batch no. 195.7 bought from Rice University) were added to 2 mL of Milli-Q water (Millipore, 18.2 MΩ) containing one weight percent (1 wt %) of F108 ((C_5_H_10_O_2_)_n_, average molecule weight 14,600 Da). The mixture was homogenized and sonicated with a tip sonicator (Misonix-XL2000, 6 W output) for 10 s. The formed dispersion was then centrifuged (eppendorf, centrifuge 5804 R) at 10,000 rpm for 60 min at 4 °C to remove large aggregates and bundled nanotubes. The supernatant (upper ~70–80% dispersion) was transferred to a clean glass vial and stored at room temperature for further use. Brij35- and Tween20-coated SWCNTs were prepared using an equivalent protocol. Absorption spectrum was recorded for every suspension with a Cary 5000 instrument in a range from 400 nm to 1400 nm. Spectroscopy measurements were performed on 1 mL samples in a 1-cm wide sterile cuvette at room temperature.

In situ polymerized poly(vinyl pyrrolidone) (ISPVP)-coated SWCNTs: We followed a standard protocol described in Reference [23] for preparing ISPVP-coated SWCNTs. Briefly, 1 mL of SWCNTs suspension in 1 wt % sodium dodecylbenzene sulfonate (SDBS: C_18_H_29_O_3_SNa)—prepared using the same protocol as above—was added to 3 mL of 1 wt % vinyl pyrrolidone (VP) solution to obtain VP-SDBS-SWCNTs dispersion. In this VP-SDBS-SWCNTs dispersion, the concentration of SWCNTs was around 10 μg/mL, SDBS concentration was 0.25 wt %, and VP concentration was 0.75 wt %. To achieve the in situ polymerization of VP on SDBS-SWCNT, 1 M HCl solution was added in a dropwise manner to the VP-SDBS-SWCNTs dispersion to turn the pH to 2, and the dispersion was then kept at room temperature for 30–45 min to allow VP fully polymerized on SDBS-SWCNTs, finally the pH of the dispersion was returned to 7–8 by adding 1 M NaOH solution in a dropwise manner. The dispersion was then stored at room temperature for further use.

PLPEG-coated SWCNTs: HiPco-synthesized SWCNTs were suspended by PLPEG molecules (#MPEG-DSPE-5000, Laysan Bio, Inc., Arab, AL, USA) in D_2_O. 1 mg of raw SWCNTs, and 5 mg PLPEG were added to 5 mL D_2_O water and dispersed by tip sonication (20 W output for 8 min in an ice bath). Nanotube bundles and impurities were precipitated by centrifuging the dispersion at 3000 rpm for 60 min at room temperature. The supernatant was collected and stored at 4 °C until further use. The concentration of the PLPEG-coated SWCNT solution was estimated to be 3 μg mL^−1^.

### 3.2. Cell Culture Studies

Cell culture protocol: COS-7 cells were cultured on microscope coverslips in DMEM medium supplemented with streptomycin (100 μg/mL), penicillin (100 U/mL), and 10% bovine serum in a 95% humidified atmosphere, 5% CO_2_, at 37 °C. Cells were cultured every three to four days and used up to passage 20.

Cell proliferation assay: SWCNTs suspension was first purified by filtering through a 100 kDa MWCO filter (Millipore, Burlington, MA, USA) in a centrifuge chamber (eppendorf, centrifuge 5804 R) at 5000 rpm for 30 min at 4 °C to remove excess surfactants, and the precipitation was re-suspended in PBS 1× for further application. COS-7 cells were cultured on microscope coverslips and incubated with SWCNTs at a final concentration of 1 μg/mL in DMEM medium for either one day or four days at 5% CO_2_ at 37 °C. COS-7 cells were then cleaved from the coverslips using trypsin (1%) and harvested by centrifugation at 2000 rpm for 5 min. This concentration of 1 μg/mL was chosen to be in single-molecule regime. COS-7 cells were suspended in 1 mL PBS 1×. For cell counting, using microscopy at 10× magnification, cells were diluted with a factor of 100× in PBS 1×, a drop 10 μL was put at the center of hemocytometer and covered by a coverslip. The sample was then mounted on a homemade microscope with white light illumination at room temperature and counted. Total cell number (in 1 mL suspension) = cell number on hemocytometer (counted cell number in the four outer squares of hemocytometer) × 2500 × 100 (dilution factor). The cell number was calculated and normalized by the control cell number without nanotube incubation.

Cell viability assay: COS-7 cells samples were similarly prepared by following the protocol for cell proliferation assay. In the cell counting procedure, the dead cells were stained by trypan blue (4%) for 20 min at room temperature. A 100× dilution factor was used on the microscope, the total cells and dead cell numbers were counted, viability was given by 1 − (dead cell number/total cell number). Control cells were prepared by following the same protocol, but without SWCNTs administration. The cell number was calculated and normalized by the control cell number without nanotube incubation.

### 3.3. Single Nanotube Fluorescence Microscopy Setup

Nanotubes were excited by a tunable Ti:Sa laser to preferentially excite (6,5) SWCNTs at the resonance of the dark K-momentum exciton. The beam was focused into the back aperture of a high NA objective (60×, NA 1.0) mounted on an upright microscope (Nikon, Tokyo, Japan), with an excitation intensity of 10 kW/cm^2^ of circularly polarized light at the sample. The fluorescence was collected with the same objective and imaged on a low noise EMCCD camera (Roper Scientific SAS, Evry, France) to produce wide-field images of individual SWCNTs. A dichroic mirror (FF875-Di01, Semrock, Rochester, NY, USA) and the combination of long- and short-pass emission filters (ET900LP, Chroma Technology Corp., Bellows Falls, VT, USA; FESH1000, Thorlabs SAS, Maisons-Laffitte, France) were used in order to illuminate and detect the (6,5) SWCNTs’ emitted fluorescence. Images of SWCNTs were recorded with 30 ms integration time per frame.

### 3.4. Agarose Sample Preparation

Purified agarose (Sigma-Aldrich, St. Louis, MO, USA, low gelling temperature) was used without further purification. Agarose gels were prepared by adding 1 mL of Milli-Q water to 15 mg agarose powder (1.5 wt % agarose gel concentration) in a glass vial. Small nanotube solutions were mixed in the agarose preparation. For observation under the wide-field microscope, 80 µL of agarose solution mixed with nanotubes were plated between a glass coverslip and a slide and sealed using vacuum grease.

## 4. Conclusions

We have shown here that PLPEG- and F108-coated SWCNTs have negligible acute (i.e., one to four days) cellular cytotoxicity at 1 μg/mL and minimal cellular interaction in comparison with several other widely used “biocompatible” surface coatings. In biological medium, PLPEG-coated carbon nanotubes display brighter luminescence than F108-coated SWCNTs, minimal unspecific interaction with cellular structures, and can be imaged at video rate for several minutes at the single tube level while diffusing in a complex aqueous network. Our work establishes that, among the main coatings currently used for SWCNT imaging, PLPEG represents the optimal coating for single nanotube tracking applications in complex biological samples.

## Figures and Tables

**Table 1:** Some known biological effects of nanotubes encapsulated with the coating used in this study.

Nanotubes	Surfactant	Biological System	Dose	Exposure Time	Assay Method	Conclusion	Reference
HiPco SWCNTs	PLPEG	Human serum and intravenous injection in rats	60 μg/mL	0.5 h	ELISA	Activation of the complement system by SWCNTs in undiluted normal human serum and in vivo rats.	[18]
HiPco SWCNTs	PLPEG	Intravenous/brain injection in rats	60 μg/mL	0.5 h to days	Fluorescence	In vivo SWCNT circulation (vascular system, brain). Stable imaging in vivo and in tissues.	[11,17]
HiPco SWCNTs	Pluronic F108	J774.1A mouse peritoneal macrophage	11 ng/mL	0, 8, 18 and 24 h	Fluorescence	Macrophages can ingest significant quantities of SWCNTs without showing toxic effects. Stable imaging in cells.	[19]
HiPco SWCNTs	Pluoronic F127	HeLa cells	200 μg/mL	2 days	Fluorescence imaging	Induction of actin bundling in cells, reduced cellular proliferation.	[20]
Arc Discharge SWCNTs	Tween20	Pathgen free guinea pigs	50 mg/mL	4 weeks	Lung function, bronchoalveolart lavega	No abnormalities of pulmonary function or measurable inflammation in guinea pigs.	[21]
MWCNTs	Sterile saline + Brij 35	Incubation with cytochrome P450 enzymes (CYP3A4)	0.067 mg/mL	5 min at 37 °C	Capillary electrochromatography, enzyme activity monitoring	No effect on CYP3A4 activity. Substantial improvement of migration time and peak shape repeatability in capillary electrochromatography.	[22]
HiPco SWCNTs	ISPVP	Human embryonic kidney cells (HEK cells)	1/30 μg/mL	5 min at RT or 12 h at 37 °C	Fluorescence	Stable imaging in cells.	[23]

HiPco: High-Pressure carbon monoxide; SWCNTs: Single-walled Carbon Nanotubes; MWCNTs: Multi-walled Carbon Nanotubes; PLPEG: Phospholipid-polyethylene Glycol; ISPVP: In Situ Polymerized (poly)vinyl pyrrolidone.
